# Assessment of Nuclear Fusion Reaction Spontaneity via Engineering Thermodynamics

**DOI:** 10.3390/e26100884

**Published:** 2024-10-21

**Authors:** Silvano Tosti

**Affiliations:** Nuclear Department, ENEA, Via E. Fermi 45, 00044 Frascati, Italy; silvano.tosti@enea.it

**Keywords:** nuclear fusion, thermodynamics, chemical potential, entropy

## Abstract

This work recalls the basic thermodynamics of chemical processes for introducing the evaluation of the nuclear reactions’ spontaneity. The application and definition of the thermodynamic state functions of the nuclear processes have been described by focusing on their contribution to the chemical potential. The variation of the nuclear binding potentials involved in a nuclear reaction affects the chemical potential through a modification of the internal energy and of the other state functions. These energy changes are related to the mass defect between reactants and products of the nuclear reaction and are of the order of magnitude of 1 MeV per particle, about six orders of magnitude larger than those of the chemical reactions. In particular, this work assesses the Gibbs free energy change of the fusion reactions by assuming the *Q_value_* as the nuclear contribution to the chemical potential and by calculating the entropy through the Sackur–Tetrode expression. Then, the role of the entropy in fusion processes was re-examined by demonstrating the previous spontaneity analyses, which assume a perfect gas of DT atoms in the initial state of the fusion reactions, are conservative and lead to assessing more negative ΔG than in the real case (ionized gas). As a final point, this paper examines the thermodynamic spontaneity of exothermic processes with a negative change of entropy and discusses the different thermodynamic spontaneity exhibited by the DT fusion processes when conducted in a controlled or uncontrolled way.

## 1. Introduction

Nuclear fusion is considered an inexhaustible and inherently safe energy source, and its exploitation will contribute to achieving the sustainable development goals needed to face the challenges raised by the climate change trend [1,2,3,4,5]. Despite the large and competent efforts spent by the nuclear fusion research communities over the last 70 years, the realization of fusion power plants has faced significant hurdles related to the development of specific technologies and materials and to the control of the magnetized plasma instability [6,7]. In recent years, significant investments from private enterprises and new public–private partnerships accelerated the development of viable technologies required for the exploitation and commercialization of fusion power plants [8,9,10].

In this work, fusion nuclear processes are studied via a thermodynamic approach like that adopted to investigate the chemical processes. The assessment of the state functions (e.g., *U*, *H*, *S*, *G*, *A*) allows for the evaluation of the spontaneity of a process and is useful to preliminary design the main process units of the chemical plants (e.g., reactors, heat exchangers, separators, etc.). This engineering thermodynamic approach is proposed to investigate the feasibility of fusion processes and is compared with the nuclear physics criteria so far adopted. In particular, the main state functions and their application to closed, open and isolated systems are first discussed by underlining the similarities between the thermodynamic analysis of the chemical and nuclear processes. Then, the concept of chemical potential is discussed for the nuclear reactions, where it accounts for the contribution to the internal energy (*U*) of the nuclear energy associated with the nuclear forces’ potentials.

Preliminary works evaluating the spontaneity of nuclear reactions, both fusion and fission, via engineering thermodynamics have shown the different roles of the entropy in fission (Δ*S* > 0) and fusion (Δ*S* < 0) nuclear reactions [11]. Negative changes in the entropy of the D-based reactions occurring in tokamaks have been assessed by applying the H-function theorem to both neutrons and charged particles (deuterium, tritium, He3 and protons) [12] and with the Sackur–Tetrode theory [13]. The entropy changes of selected D-based fusion reactions of interest for the exploitation of fusion energy in tokamak machines have been calculated via the Sackur–Tetrode equation while the contribution of the nuclear bonds to the changes of the chemical potential has been accounted for by the change of enthalpy calculated through the energy released (*Q_value_*). The feasibility of these processes at the temperature of the hot plasmas (10^8^ K) was assessed taking into account the reaction rates and the change of Gibbs free energy [13]. In the fusion processes, a share of the energy made available by the reaction is spent to balance the entropic term (-*T* Δ*S*) so that, at a very high temperature, they become non-spontaneous. The results confirmed that, among the D-based reactions carried out in the tokamak reactors, the DT one is the most promising, being characterized by both high thermodynamic spontaneity (Δ*G* = −16 MeV) and appropriate reactivity (about 2 × 10^−22^ m^3^/s).

Through the discussion of the thermodynamic functions and reaction systems modeling, this paper examinates the hypotheses made in the previous thermodynamic analyses of nuclear fusion systems and, especially, the assumption of considering at the initial state of the D-based reactions a perfect gas of DT atoms instead of a plasma (ionized gas).

Finally, the parallel between the thermodynamics of the exothermic chemical reactions characterized by Δ*S* < 0 (e.g., the water formation) and the DT fusion process is exploited for debating the different thermodynamic spontaneity exhibited by reactions with negative entropy changes when conducted in a controlled or uncontrolled way.

## 2. The First and Second Laws of Thermodynamics

In thermodynamics, a system could be defined by how it interacts with its surrounding environment [14,15]. In this view, three kinds of systems are identified:

- The closed systems that can exchange energy (work or heat) with the ambient while no exchange of matter is allowed;
- The open systems that can exchange both energy and matter with the ambient;
- The isolated systems that cannot exchange energy or matter with the ambient.

An open system is delimited by a control volume, i.e., a fixed region in space that allows mass and energy to move across the boundary between the system and its surroundings.

It is useful to introduce the concept of “universe” as the union of the studied system and its surroundings. The universe is intrinsically an isolated system not capable of exchanging matter and energy with its outside.

The First law of thermodynamics consists of an energy balance:

*dE* = *δQ* − *δL* (1)

where *dE* is the change in the energy content of the system and *δQ* and *δL* represent the heat and the work exchanged with the outside. In particular, the heat entering the system and the work performed by the system are positive.

Heat (*Q*) and work (*L*) are not properties of the system; they are called path functions because their magnitude depends on the states and the specific process path. Their differential is not exact and is indicated by *δ*. Differently, the amount of energy of a system is a property of the system; it is a state function, meaning that (i) its magnitude depends on the state only, not on the reaction path followed, and (ii) its differential (*dE*) is exact.

### 2.1. Entropy

The entropy is a state function defined as follows:

(2) $$dS=\frac{\delta Q}{T}+dS_{i}$$

where δQ is the heat exchanged by the system with the outside and *dS_i_* is the contribution to the change in entropy due to the irreversibility of the reaction. In general, *dS_i_* ≥ 0, while *dS_i_* = 0 in reversible processes. This definition embodies one of the enunciations of the Second law and, as discussed hereafter, will be exploited in the analysis of the reactions’ spontaneity.

In isolated systems where ${\delta Q=0\;and\;dS=dS}_{i}$, the entropy of irreversible processes can only increase, while in the reversible processes, there is no change of entropy. That is, in isolated systems, it results:

Δ*S* > 0       for irreversible processes (3)

Δ*S* = 0     for reversible processes (4)

The above Formulas (3) and (4) are valid for the thermodynamic analyses of the “universe” that, as above defined, is considered an isolated system.

### 2.2. Energy Balances in Thermodynamic Systems

First, the main state functions of interest for the thermodynamic analyses are introduced by assuming that there is no composition change of the components of the system, i.e., no chemical reactions or other matter transformations are taking place.

The discontinuous processes, for instance, those occurring in batch operations of chemical plants, can effectively be represented by closed systems. For these processes, the energy balance (1) is rewritten as follows:

*dE* = *δQ* − *δL_c_* (5)

where *L_c_* is the amount of the work exchanged by a closed system.

Generally, the thermodynamic analyses consider only the energy at a microscopic level, while the contribution coming from the potential and kinetic energy at the system’s macroscopic level is neglected. The energy at a microscopic level is represented by *U*, the internal energy. It takes into account several contributions, such as the kinetic (e.g., translation, rotation) energy of the particles (molecules, atoms, etc.) and the chemical energy related to the molecular forces’ potentials. Afterwards, we can see that in the case of nuclear reactions, the internal energy regards the energy associated with the nuclear forces’ potentials, too. Once the internal energy is introduced, the Formula (1) can be rewritten:

*dU* = *δQ* − *δL_c_* (6)

Under the further assumption of reversible processes, for a closed system we have:

(7) $$dS=\frac{\delta Q}{T}$$

and since, for such systems, the only form of work depends on volume changes:

*δLc* = *p dV* (8)

and then:

*dU* = *TdS* − *p dV* (9)

Although such an expression is commonly proposed to describe the exchange of work and heat of a closed system, in case transformations of matter occur (e.g., in the presence of chemical or nuclear reactions) this formula has to be corrected as will be discussed in the following Section 2.3.

From (9), it results that for reversible processes the internal energy change of closed systems, (*TdS* = *δQ*) corresponds to the heat exchanged at a constant volume (*Q_V_*):

*ΔU* = *Q_V_* (10)

A continuous process is realistically modeled by an open system: in such a case, the energy balance represented by Equation (1) must take into account the energy of the fluid streams entering and leaving the control volume. This energy is given by the product “*pV*” and, consequently, Equation (1) becomes:

*d* (*pV*) + *dU* = *δQ* − *δL_o_* (11)

where *dL_o_* indicates the work exchanged in an open system. For such a system, it is convenient to introduce the enthalpy, a state function expressed by:

*H* = *U* + *pV* (12)

and therefore:

*dH* = *TdS* + *V dp* (13)

Combining the above formulas, it results:

*δL_o_* = *V dp* (14)

and the change of enthalpy in open systems with reversible processes corresponds to the heat exchanged at a constant pressure (*Q_p_*):

Δ*H* = *Q_p_* (15)

In thermodynamics, the spontaneity of a process is put in relationship with the maximum work that can be performed. This quantity is called free energy and is described through two state functions. The first one is the Gibbs free energy:

*G = H* − *TS* (16)

In line with the previous discussion, it results:

*dG* = −*S dT* + *V dp* (17)

The second one is the Helmholtz free energy, which is expressed by:

*A* = *U* − *TS* (18)

that is:

*dA* = −*S dT* − *p dV* (19)

Based on expressions (17) and (19), the equilibrium conditions of reversible processes are stated by Δ*G* = 0 when operating at a constant temperature and pressure and by Δ*A* = 0 when operating at a constant temperature and volume.

### 2.3. Presence of Matter Transformations: The Chemical Potential

The internal energy (*U*) and the other state functions (*H*, *G*, *A*, *S*) are extensive functions that can vary because of:

- A matter exchange with the surroundings (this is the case of open systems);
- A composition change of the system’s components (this can happen in both open and closed systems due to the presence of transformations of matter within them).

Relationships (1) to (19) have to be further modified when a matter transformation occurs in the system. In fact, when chemical reactions take place, the change of chemical compositions of the system components is related to a variation of the molecular forces potentials, which, in turn, involves the change of the internal energy and of the other state functions defined above. For chemical reactions, the order of magnitude of these energy changes is 1 eV per particle.

In a system comprising “c” components (1, 2, 3, …c), each one consisting of a certain number of particles (*n*_1_, *n*_2_, *n*_3_, *… n_c_*), the above-defined state functions can be represented as:

*U* = *U* (*S*, *V*, *n*_1_, *n*_2_, *n*_3_, … *n_c_*) (20)

*H* = *H* (*S*, *p*, *n*_1_, *n*_2_, *n*_3_, … *n_c_*) (21)

*G* = *G* (*T*, *p*, *n*_1_, *n*_2_, *n*_3_, … *n_c_*) (22)

*A* = *A* (*T*, *V*, *n*_1_, *n*_2_, *n*_3_, … *n_c_*) (23)

The differential of the internal energy is:

(24) $$dU={\left(\frac{\partial U}{\partial S}\right)}_{V,\;n_{i}}+{\left(\frac{\partial U}{\partial V}\right)}_{S,\;n_{i}}+\sum_{i=1}^{i=c}{\left(\frac{\partial U}{\partial n_{i}}\right)}_{S,\;V,n_{j\neq i}}dn_{i}$$

This formula precisely identifies the contribution to the internal energy change coming from the chemical transformations. In fact, at constant *V* and *S*, it is:

(25) $$dU=\sum_{i=1}^{i=c}{\left(\frac{\partial U}{\partial n_{i}}\right)}_{S,\;V,n_{j\neq i}}dn_{i}$$

The term:

(26) $$\mu_{i}={\left(\frac{\partial U}{\partial n_{i}}\right)}_{S,\;V,n_{j\neq i}}$$

is called the chemical potential of the component “*i*” and corresponds to the amount by which the capacity of the phase/system for performing work (other than the work of an expansion) is increased per unit amount of substance added [15].

Similar relationships between the chemical potential and the above-defined state functions can be obtained:

(27) $$\mu_{i}={\left(\frac{\partial U}{\partial n_{i}}\right)}_{S,\;V,n_{j\neq i}}={\left(\frac{\partial H}{\partial n_{i}}\right)}_{S,\;p,n_{j\neq i}}={\left(\frac{\partial G}{\partial n_{i}}\right)}_{T,\;p,n_{j\neq i}}={\left(\frac{\partial A}{\partial n_{i}}\right)}_{T,\;V,n_{j\neq i}}$$

The chemical potential is very useful to discuss the behavior of (i) open systems and (ii) closed ones in which changes of compositions take place. For the component “*i*” and under appropriate operating conditions, the chemical potential is calculated as the partial derivative of a state function with respect to its number of moles. The derivative of the internal energy is conveniently taken into consideration in processes at constant entropy and volume, the derivative of enthalpy in those at constant entropy and pressure, the derivative of the Gibbs free energy in those at constant temperature and pressure and the derivative of the Helmholtz free energy in those at constant temperature and volume.

Introducing the chemical potential, expressions (17) and (19) can be rewritten:

(28) $$\mathrm{dG}=-S\mathrm{dT}+\;+V\mathrm{dp}+\sum_{1}^{c}\mu_{i}\;{dn}_{i}$$

(29) $$\mathrm{dA}=-S\mathrm{dT}-p\mathrm{dV}+\sum_{1}^{c}\mu_{i}\;{dn}_{i}$$

This means that at constant temperature and pressure (or temperature and volume), the achievement of equilibrium conditions Δ*G* = 0 (or Δ*A* = 0) requires that no chemical and nuclear reactions occur, that is $\sum_{1}^{c}\mu_{i\;}{dn}_{i}$ = 0.

In general, in classical thermodynamics, the chemical potential does not take into account the presence of nuclear reactions, i.e., it disregards the contribution coming from the new nuclear bond potentials established as a consequence of the nuclear processes. The energy changes related to the energy released during the nuclear process, the *Q_value_*, correspond through Einstein’s equation to the mass defect between reactants and products of the reaction. In the following, it will be introduced how to evaluate the nuclear reactions’ contribution to the chemical potential, a term that is of the order of 1 MeV per particle, about a factor 10^6^ higher than that of the chemical reactions.

## 3. Spontaneity of Chemical Reactions

In engineering thermodynamics, the spontaneity level of a chemical reaction is expressed in terms of the maximum work that can be performed. Since the chemical processes are usually evaluated at a constant pressure and temperature, their spontaneity is commonly studied through the assessment of the change of the Gibbs free energy.

The change of Gibbs free energy represents the amount of energy made available to produce work and corresponds to the energy released as the heat (Δ*H* = *Q_p_*) net of the entropic contribution (*T* Δ*S*). A reaction is spontaneous when Δ*G <* 0 and non-spontaneous when Δ*G >* 0, while the condition Δ*G* = 0 indicates that the reaction is at equilibrium.

At a constant pressure and temperature, from the expression (16), it results:

Δ*G* = Δ*H* − *T* Δ*S* (30)

The Δ*H* and Δ*S* of the chemical reactions can be evaluated from the values of the enthalpy and entropy of the reactants and products (molecules and atoms) that are calculated, in turn, from those of the pure substances available in the chemistry and physics databases. Alternatively, statistical physics proposes methods to calculate the thermodynamic functions [15,16].

### 3.1. Statistical Analogous of Free Energy and Entropy of a Monoatomic Perfect Gas

Statistical mechanics calculate the thermodynamic properties of a system from a knowledge of its partition function [15,16]. For a perfect gas, the molecular partition function is made of two parts related to the translational and the internal motion of the molecules. The internal state is, in turn, the sum of two terms due to the electronic excitations and the combined rotational and vibrational motion. For a monoatomic gas, the energy is entirely translational and the comparison of the statistical analogous with thermodynamic properties leads to the following expression for the Gibbs free energy:

(31) $$G=-n\cdot R\cdot T\;\cdot \left[ln\left(\frac{M^{\frac{3}{2}}\cdot T^{\frac{5}{2}}}{P}\right)-3.16\right]$$

where *n* is the number of moles (1 mole = 6.022 × 10^23^ particles), *P* is the pressure (atm), *M* the molecular weight, *T* the temperature (K) and *R* the gas constant.

This theory takes into account the translational and internal energies of the molecules and cannot be applied when nuclear reactions occur. As described in Section 4.3, the change in the Gibbs free energy of nuclear reactions could be derived by a reworking of Formulas (16) and (30), where the contribution of the energies corresponding to the change of the nuclear bond energies is given by Δ*H* through its relationship with the *Q_value_*.

Moving from Formula (31), it is possible to obtain the expression of the entropy since, for a monoatomic gas, it is:

(32) $$H=\frac{5}{2}\;R\;T$$

Combining (31) and (32) with Formula (16), results in the well-known Sackur–Tetrode equation:

(33) $$S=n\cdot R\cdot \left[ln\left(\frac{M^{\frac{3}{2}}\cdot T^{\frac{5}{2}}}{P}\right)-1.16\right]$$

It is notable to discuss the validity of this expression for calculating the entropy in the case of nuclear reactions. In classical thermodynamics, entropy is associated with the way the energy is exchanged and not with the energy content of the system; such a concept corresponds to that of statistical thermodynamics, which consider entropy as a measure of the number of ways a system can be arranged and describes the entropy as a term related to the configuration of the system [15,16]. In this view, the Sackur-Tetrode Equation (33) can be applied to calculate the entropy of the reactants and products of nuclear reactions.

### 3.2. Exothermic Reactions with Negative Change in Entropy: The Example of Water Formation

As an example of an exothermic reaction with Δ*S* < 0, let us consider the reaction between hydrogen and oxygen to produce water:

*H*_2_ + 0.5 *O*_2_ => *H*_2_*O* (34)

This is a strongly exothermic reaction (Δ*H* = −250 kJ/mol at 25 °C) that takes place with a reduction of the mole number and, in fact, is characterized by a negative change of entropy (Δ*S* = −55 J/mol at 25 °C). In the following equation, Δ*S_r_* will indicate the change of entropy of reaction (34).

**Figure 1:**
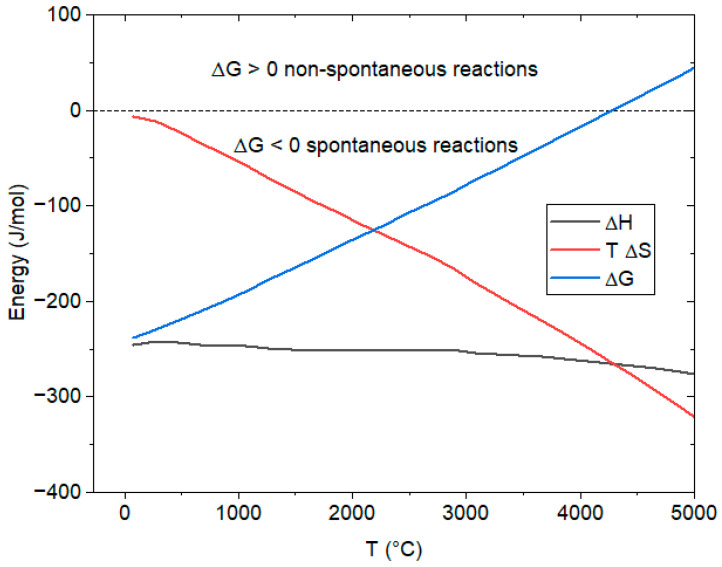
Change of the state functions vs. temperature for the water formation reaction (34): results from simulation [17].

As shown by the graph of Figure 1 obtained by a simulation [17], the variation of Δ*H* with the temperature is modest, while the entropic term “*T* Δ*S_r_*” increases by about 55 kJ/mol for every 1000 K of temperature increase. Regardless of its enormous exothermicity, this reaction becomes non-spontaneous (i.e., Δ*G* > 0) when the temperature is slightly over 4000 K. This temperature is approximately given by:

T* ≈ Δ*H*/Δ*S_r_* (35)

After the study of its thermodynamic behavior, to comprehensively model the system where the reaction takes place, it is necessary to establish if it occurs in a controlled or uncontrolled way.

For instance, the huge heat released by reaction (34) can be exploited in a controlled process represented by an oxyhydrogen torch used to cut materials. On the other hand, the same reaction could be responsible for uncontrolled processes, such as the hazardous explosions of hydrogen storage systems.

**Figure 2:**
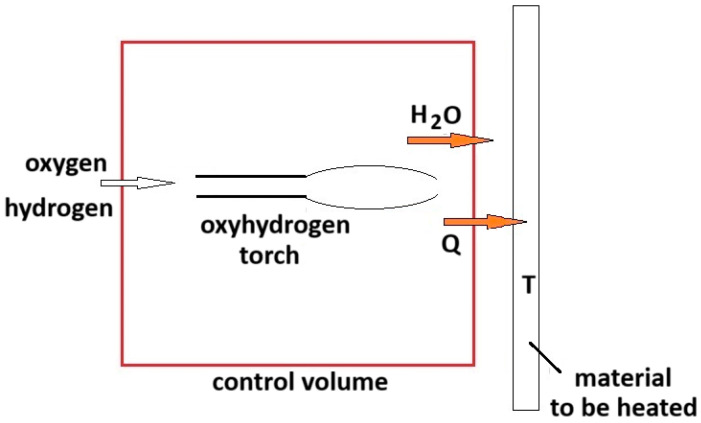
Modeling of the water formation reaction performed by an oxyhydrogen torch.

Once steady-state conditions are achieved, the process exploited by an oxyhydrogen torch is nearly continuous and controlled. Under these conditions, the process can therefore be modeled as an open system defined by the control volume colored in red in the scheme of Figure 2, where an oxyhydrogen torch is used to heat and cut a metal sheet. Under the assumption of quasi-steady-state conditions, the heat (*Q*) is transferred from the torch to the material, which remains at enough constant temperature (*T*), e.g., around its melting point. For temperatures below T*, the term |*T* Δ*S_r_*| is smaller than the absolute change of enthalpy and reaction (34) proceeds spontaneously; in fact, it results that Δ*G* = Δ*H* − *T* Δ*S* < 0, which is the condition stating the reaction spontaneity according to the discussion at the beginning of Section 3. By considering the constant temperature of the material to be cut and neglecting the occurrence of other reactions (mixing, changes of states, etc.), the entropy change of the surroundings is a positive term given by the sum of the term “heat exchanged divided by the temperature” and the term due to the irreversibility:

(36) $$\Delta S_{\mathrm{sur}}=\frac{Q}{T}\;+\;dS_{i}$$

The entropy change of the “universe”, that is, the union of the open system defined by the control volume and its surroundings, results:

Δ*S_univ_* = Δ*S_r_* + Δ*S_sur_* (37)

The entropy of the universe (an isolated system) can only increase (Δ*S_univ_* ≥ 0) and, in fact, below T*, the term Δ*S_sur_* (positive) balances the term Δ*S_r_* (negative). If the heat exchange takes place reversibly, it results that Δ*S_i_* = 0 and Δ*S_r_* = Δ*S_sur_*, corresponding to the condition Δ*H* = *Q*.

For *T* < T*, the process occurs spontaneously, i.e., the heat released is larger than the energy spent to make the reaction system more ordered. The entropy reduction occurring in the system is balanced by the entropy increase of the outside so that the entropy change of the universe (system + surroundings) is positive (irreversible process) or at least null (reversible process).

It is remarkable to see what happens when the same reaction (34) takes place in processes evolving in an uncontrolled way, e.g., the explosions of a hydrogen storage system. Once triggered by modest energy activation values, such a process is always spontaneous; in the presence of a leak, a small spark is enough to initiate the blast of a hydrogen storage vessel. In general, it is not easy to model these processes; the composition of the gas mixture at the time of ignition and after the explosion could not be clearly known and, therefore, the initial and final states are not precisely defined. Furthermore, the mixing of the chemical species during these processes implicates an increase of entropy, which is not clearly computable [18]. Let us consider a finite volume, in principle as small as possible, including the hydrogen storage vessel and a limited region of space all around the vessel. Since the explosion occurs very quickly, the surroundings outside the control volume are practically unaffected by the explosion. There is no time to exchange heat and matter through the boundary of the finite volume that therefore identifies an isolated system in which, according to (3) and (4), the entropy can only increase (Δ*S* ≥ 0). In fact, the contribution to the entropy increase coming from the diffusion and mixing of gas molecules (parallel reactions) balance the negative entropy change of the main reaction, (34).

### 3.3. Controlled vs. Uncontrolled Processes

As seen in continuous and controlled processes consisting of a unique chemical reaction characterized by Δ*H* < 0 and Δ*S_r_* < 0, a part of the energy released is spent to make order. The feasible exploitation of a process requires that it be managed in a “controlled” and “orderly” manner. The thermodynamics through the Second law poses a limit to the share (*T* Δ*S_r_*) of free energy (Δ*G*) that can be spent to make order: such a limit is given by the heat released to the outside (*Q*) that determines the increase of the entropy of the surroundings (Δ*S_sur_* = *Q*/T) that, in turn, has to overcome the absolute value of the entropy change of the system where the controlled process occurs (|Δ*S_r_|*). As can be seen, this condition translates into the existence of a critical temperature (T* = Δ*H/*Δ*S_r_*) below which the process proceeds spontaneously.

In uncontrolled processes, the same exothermic reaction (with Δ*H* < 0 and Δ*S_r_* < 0) proceeds in parallel with other reactions characterized by an increase of the entropy (Δ*S* > 0), e.g., the diffusion and mixing of different species. This positive entropy change may overcome that of the main reaction so that the overall entropy change results as positive, and the process could be always spontaneous regardless of the temperature. In Table 1, the thermodynamic spontaneity of the processes characterized by a main reaction with Δ*S*_r_ < 0 and Δ*H* < 0 is reported by distinguishing between controlled and uncontrolled processes.

## 4. Feasibility Analysis of Nuclear Reactions

The distribution of the binding energy per nucleon vs. the mass numbers (A) of the atoms is used for distinguishing between fusion and fission reactions. Positive mass defects occur in fusion reactions among light atoms with a mass number (A) lower than 56 and in fission reactions where larger atoms (with A over 56) split into smaller fragments. The mass defect, that is, the difference between the mass of the products and the reactants of a nuclear reaction, corresponds, through the Einstein’s equation, to energy release and is defined by the *Q_value_*.

Since most of the energy released from a nuclear process is in the end changed into heat, the *Q_value_* could be linked to the change of enthalpy. A positive *Q_value_*, indicating that the nuclear reaction is releasing heat corresponds to a negative enthalpy change:

*Q_value_* = −Δ*H* (38)

As discussed above, the enthalpy change (|Δ*H*|) of chemical reactions is associated with the particles’ kinetic energy and to the potentials of the molecular forces; therefore, it is significantly lower (≈1 eV) than that of the nuclear reactions (≈1 MeV) associated with the potentials of the nuclear forces. In this view, the *Q_value_* is related to the contribution to the chemical potential of the nuclear reactions.

### 4.1. Comparison Between Chemical and Nuclear Reactions

In nuclear processes, the presence of a positive *Q_value_* is one of the criteria adopted for establishing their spontaneity together with the verification of the reaction rate, a parameter related to the reaction probability. This approach is similar to that adopted to verify the feasibility of a chemical reaction in which the Gibbs free energy change is assessed together with the reaction kinetics. In fact, the energetic aspects (namely, the *Q_value_* for the nuclear processes and the Δ*G* for the chemical ones) consider the spontaneity of a reaction regardless of its speed and, in principle, are valid for studying what happens at equilibrium (i.e., at infinite time) without providing any info about the reaction times. For instance, a chemical reaction characterized by a negative Δ*G* could proceed very slowly due to the presence of intermediate reaction steps needing high energy barriers to be overcome. The chemical kinetics studies the reaction steps and their energy barriers; it assesses the reaction rates as well as the capability of the catalysts to modify the reaction patterns and speed up the processes. In analogy, after the assessment of the *Q_value_*, the feasibility analysis of nuclear processes has to consider their reaction kinetics. In fusion reactions, positively charged atoms must come into contact by winning the repulsive Coulomb force, and then their reaction rate is related to the probability of tunneling the corresponding potential barrier according to Gamow’s theory [19]. The reaction rate of fusion reactions could be calculated through models taking into account the energy distribution of the particles [20,21,22].

### 4.2. Spontaneity of Nuclear Fusion: The Role of Entropy

So far, the spontaneity of a nuclear reaction has fundamentally been linked to the amount of energy released (*Q_value_*), while the evaluations of the changes in Gibbs free energy (Δ*G*) and entropy (Δ*S*) have been neglected.

In previous works, the role played by entropy in fission and fusion reactions has been introduced through engineering thermodynamics [11,13]. This thermodynamic analysis moved from the qualitative evaluation that the change in entropy is positive for fission reactions where “heavy nuclei split into smaller fragments”; in this way, the processes proceed with an increase of the particle number, creating a more disordered system (Δ*S* > 0). The opposite situation (Δ*S* < 0) occurs for the fusion processes where “light atoms merge to form a heavier nucleus”.

**Figure 3:**
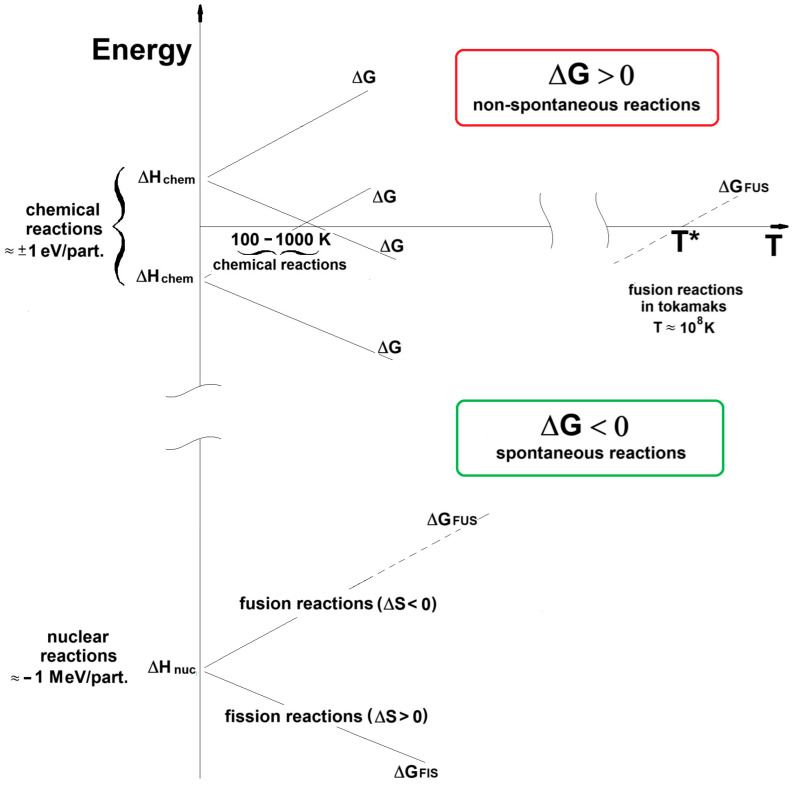
Change in Gibbs free energy vs. the temperature. Comparison of the behavior of chemical and nuclear reactions. Δ*H_chem_* and Δ*H_nucl_* indicate the change in enthalpy of chemical and nuclear reactions, respectively. Δ*G_FIS_* and Δ*G_FUS_* indicate the change in Gibbs free energy of fission and fusion reactions, respectively [13].

In Figure 3, the change in Gibbs free energy vs. the temperature of both chemical and nuclear reactions is reported. At low temperatures, the nuclear reactions exhibit values of Δ*G* in the deep negative part of the graph because of their very large negative change of enthalpy (*Q_value_* = −Δ*H* ≈ 1 MeV), well beyond those of the chemical reactions characterized by Δ*H* of the order of ± 1 eV.

According to expressions (30) and (38), for the nuclear reactions, the following results:

Δ*G* = −*Q_value_* − *T* Δ*S* (39)

The change of Gibbs free energy, i.e., the maximum work that can be performed corresponds to the *Q_value_* minus the contribution coming from the entropic term “*T* Δ*S*”.

The nuclear fission reactions have Δ*H* = −*Q_value_* and Δ*S* > 0, are always spontaneous (Δ*G* < 0) and their reaction spontaneity further increases with the temperature.

The nuclear fusion reactions behave differently from the fission ones and proceed similarly to the exothermic chemical reactions with Δ*S* < 0. In particular, the fusion reactions become thermodynamically non-spontaneous above a critical temperature defined by:

(40) $$T^{*}\;\approx \frac{Q_{value}}{\left|\Delta S\right|}$$

The term Δ*S* is of the order of 10^−3^ eV/K, a value numerically much smaller than the *Q_value_* (≈1 × 10^6^ eV); accordingly, the nuclear fusion reactions could become non-spontaneous at about 10^9^ K. When approaching very high temperatures, a large fraction (*T* Δ*S*) of the energy released (*Q_value_*) is spent to make the system more ordered so that the amount of work made available reduces in agreement with Formula (39).

### 4.3. Modeling of D-Based Fusion Reactions

The research activities of the exploitation of fusion energy are focused on the development of tokamak machines where a plasma of light atoms is magnetically confined at very high temperatures (over 10^8^ K) [5,7]. These fusion processes use the following deuterium-based reactions:

(41) H + 12H = 13He + 24n01   Qvalue = 17.586 MeV

(42) H + 12H = 12H + 13p11   Qvalue = 3.267 MeV

(43) H + 12H = 12He + 23n01   Qvalue = 4.032 MeV

(44) H + 12He = 23He + 24p11   Qvalue = 18.351 MeV

Reaction (41) between deuterium and tritium is the most promising; it is characterized by reaction rates higher than those of the other reactions by at least a factor of two.

**Figure 4:**
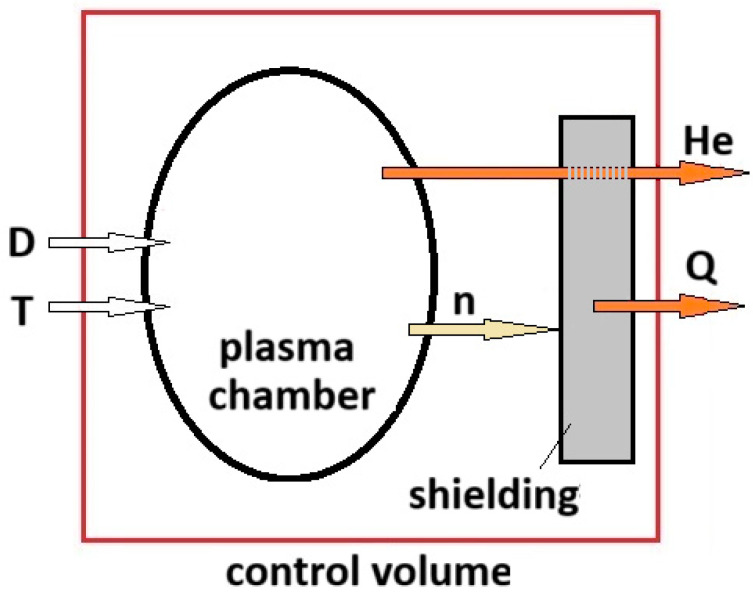
Modeling of the DT reaction carried out in a tokamak reactor.

In the present designs of nuclear reactors, the neutrons produced by the DT reaction are absorbed by the shielding and blanket systems, where their energy is changed into heat that, in turn, is used to produce electricity for the grid. More in detail, the neutrons going to the blanket interact with the Li-materials to produce tritium, which, once extracted and purified, is sent to the plasma chamber. The helium is extracted with the unburned DT fraction through the plasma exhaust system. Hereafter, to model in a simple way reaction (41), we will consider a fusion power system without a breeding blanket, as schematically represented by Figure 4. The deuterium and the tritium enter the control volume. The neutrons interact with the shielding where the heat is extracted from the control volume by a cooling system, and the helium, once separated from the plasma exhaust processing units, leaves the control volume as well.

Coherently with this scheme and adopting a formula similar to that of chemical reactions, expression (41) could be rewritten as follows:

(45) D+T=He4   ΔH=−17.59 MeV

In a first attempt, let us consider that reaction (45) involves perfect monoatomic gases under the following operating conditions:

- Initial state: deuterium and tritium at very high temperature T and low pressure (few Pa);
- Final state: He at around 700 K and atmospheric pressure.

As will be discussed in detail in the following section, the Gibbs free energy of plasma is lower than that of a perfect gas. Assuming the plasma of deuterium and tritium as a perfect gas leads to assessing more negative Δ*G* than that of the real case (ionized gas). In this way, the analysis overestimates the spontaneity level of the DT reaction (45) and is therefore conservative.

As represented by the scheme of Figure 4, only helium and heat leave the reaction system defined by the control volume; before achieving the final state, the sub-atomic particles (protons, neutrons, etc.) produced by the fusion reactions have interacted with the system’s walls and changed their energy into heat. Consequently, for the final state of reaction (45), the use of a model valid for a perfect gas at low pressure and high temperature is correct.

Formula (31) cannot be applied for the assessment of the Gibbs free energy of reaction (45) since such a formula is obtained by mechanical statistical methods, which does not take into account the contribution to the chemical potential coming from the new nuclear bond potentials established as a consequence of the nuclear processes.

Therefore, the change of Gibbs free energy of reaction (45) is assessed by Equations (16) and (38):

Δ*G* = −*Q_value_* − Δ*(T S)* (46)

The change of enthalpy through the *Q_value_* accounts for the contribution to the chemical potential coming from the new nuclear bond potentials established as a consequence of the nuclear reaction, while for the assessment of the entropy, the mechanical statistical method (Sackur–Tetrode) is justified for the analysis of nuclear reactions, as discussed in Section 3.1.

Accordingly, the Δ*G* of a tokamak where a DT plasma reacts at temperature *T*(K) and the He and the heat are recovered at 700 K has thus been calculated [13]:

(47) $$\Delta G=-Q_{value}-\left[700\;S(700)-T\;S\left(T\right)\right]$$

The kinetics of the nuclear reactions can be expressed by the reactivity, a parameter that assesses the probability of reaction per unit time and unit density of the target nuclei and is obtained by the product of the cross-section (*σ*) and the particle velocity (*v*). For the DT reaction, the average reactivity <*σ v*> evaluated considering a Maxwell particle velocity distribution was used [22,23].

**Figure 5:**
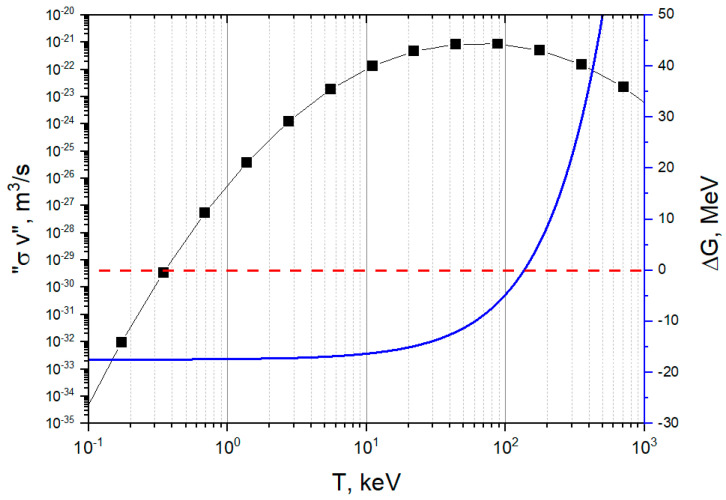
Reactivity (black line) and change of Gibbs free energy (blue line) vs. temperature for the deuterium–tritium reaction (the red dashed line indicates Δ*G* = 0; below it, the reaction occurs spontaneously) [13].

The results of this study are shown in the graph of Figure 5 [13]. The DT reaction at the tokamak machine’s temperature of 1.5 × 10^8^ K (about 13 keV) exhibits a good level of thermodynamic spontaneity (ΔG ≈ −16 MeV). At this temperature, the average reactivity is approximately about 2 × 10^−22^ m^3^/s, a value capable of guaranteeing the tritium self-sufficiency conditions of the fusion reactors [23,24,25]. The critical temperature of the DT reaction is T* ≈ 1.6 × 10^9^ K (132 keV), while its reactivity has a maximum of 7.42 × 10^8^ K (64 keV). In principle, there would be a margin to increase the temperature for improving the reactivity; however, the operation of tokamaks over 1.5 × 10^8^ K would be very challenging from an engineering point of view.

The initial state of the system consists of a plasma (an ionized gas) whose Gibbs free energy differs from that of a perfect gas, as will be examined in the next section.

### 4.4. Gibbs Free Energy of a Plasma

The previous work evaluated the entropy of the particles involved in reaction (45) under the assumption of a perfect gas behavior. As can be seen, in a good approximation, this model is coherent with the state of the reaction products (tokamak shielding or blanket systems at around 700 K and a few bars of pressure), while it is necessary to verify the applicability of this assumption to the reactants of reaction (43) consisting of a plasma.

In the presence of charged particles with a Coulomb interaction (ionized gas), the Gibbs free energy of a perfect gas is corrected as follows [16]:

(48) $$G_{plasma}=G_{id}-\frac{2\;e^{3}}{3\;T}\;{\left(\frac{\pi \;P}{N}\right)}^{\frac{1}{2}}{\left(\sum_{a}N_{a}\;z_{a}^{2}\right)}^{\frac{3}{2}}$$

where *G_plasma_* is the Gibbs free energy of the plasma, *G_id_* is that of the perfect gas, *e* is the unit charge, *N* is the number of particles, *N_a_* is the number of ions of the ath kind and *z_a_* is either positive or negative integers (so that “*z_a_ e*” denotes the charge of this particle).

**Figure 6:**
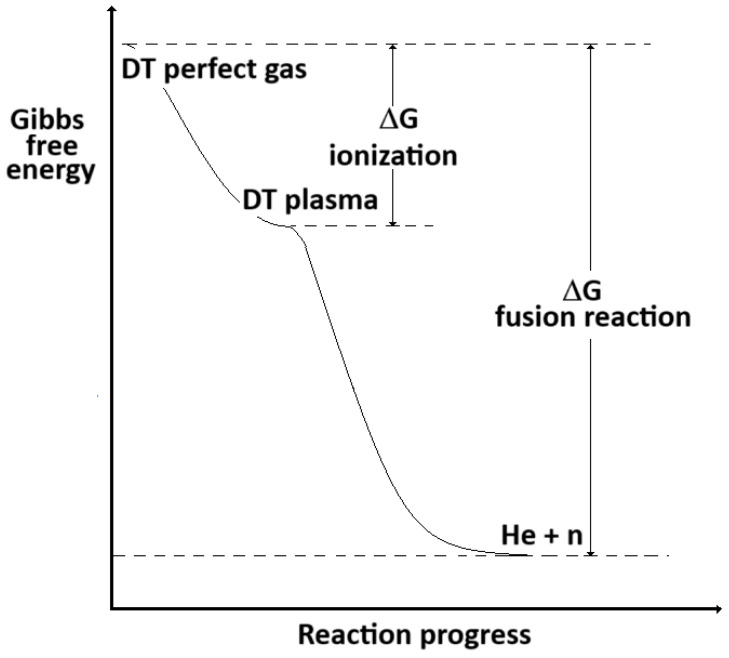
Gibbs free energy levels during the evolution of the DT reaction.

It results that *G_plasma_ < G_id_*, indicating that the ionization process *DT_id_* => *DT_plasma_* has a negative change of Gibbs free energy (*G_plasma_* − *G_id_ ≤* 0), which reduces with the temperature and approaches zero (*G_plasma_ ≈ G_id_*) for T => ∞. The Gibbs free energy levels during the evolution of the DT reaction (45) are represented in Figure 6.

According to the previous works’ results [11,13], by increasing the temperature, the Δ*G* of the fusion reaction increases, equals zero when the critical temperature is achieved (*T* = T*) and becomes positive (non-spontaneous reaction) over this temperature. Being that *G_plasma_* − *G_id_ ≤* 0, by assuming at the initial state of the fusion reactions a perfect gas of DT atoms instead of a plasma, it results that the spontaneity analysis is conservative. In fact, they lead to assess more negative Δ*G* than the real case (ionized gas) and, therefore, overestimate the spontaneity level of the DT reaction.

### 4.5. Nuclear Fusion: Controlled vs. Uncontrolled Processes

As discussed for the thermodynamic analysis of chemical reactions, the exothermic processes with Δ*S* < 0 when carried out in a controlled way proceed spontaneously only at temperatures below a critical value T* = Δ*H*/Δ*S*. These same reactions performed in an uncontrolled way see the presence of parallel processes characterized by a positive change in entropy as the diffusion and mixing of particles. According to Table 1, uncontrolled exothermic processes with Δ*S* < 0 could be spontaneous at temperatures higher than T* or be ever spontaneous. It is noteworthy to discuss how these concepts can be extended to the nuclear reaction assessment.

A clear example of an uncontrolled fusion process is represented by the explosion of a thermonuclear fusion weapon (H-bomb), and, in this case, the spontaneity of the process is unfortunately verified.

Diversely, in a power plant where the DT plasma is magnetically confined, the fusion reactions are in principle carried out in a quite controlled way; in this case, as discussed, their thermodynamic spontaneity is verified below the critical temperature T*. 

## 5. Conclusions

The main thermodynamic functions and their application to closed, open and isolated systems have been discussed by focusing on the similarities between the spontaneity analyses of chemical and nuclear processes. The concept of the chemical potential of the nuclear reactions has been discussed in relationship with the nuclear binding potentials whose variation involves a modification of the internal energy and of the other state functions. These energy changes are related to the mass defect between reactants and products of the nuclear reaction and are of the order of magnitude of 1 MeV per particle, about a factor 10^6^ larger than those of the chemical reactions.

The thermodynamic analysis suggests that (i) the higher the temperature, the larger the share (-*T* Δ*S*) of the energy released (*Q_value_*) that is spent to make the system more ordered; (ii) the amount of work that can be performed (-Δ*G*) reduces with the temperature and goes to zero approaching the temperature T*. In other words, at very high temperatures, a share of the *Q_value_* is consumed to balance the entropic term (-*T* Δ*S*), thus reducing the enormous amount of energy made available by a nuclear fusion process. Such an aspect could have a non-negligible impact on the energy efficiency of the fusion systems and should be considered in future tokamak designs.

Further, the role of entropy in fusion processes has been re-examined by demonstrating that the hypotheses related to the definition of the initial state of the fusion processes consisting of a perfect gas instead of a hot plasma are conservative and lead to overestimating the spontaneity level of the D-based reactions.

Finally, this paper compared the thermodynamic spontaneity of exothermic processes with a negative change of entropy when carried out in “controlled” and “uncontrolled” ways. These considerations could support future studies on how the operation modes of the tokamaks, namely the pulsed vs. steady state reactors, affect the spontaneity of the fusion processes.

Moreover, the results of this theoretical approach could support future work on the evaluation of tokamak systems through a thermodynamic analysis realistic enough to include the specific mass and energy balances of the entire fusion fuel cycle.

## Figures and Tables

**Table 1:** Thermodynamic spontaneity of exothermic processes with negative change of entropy when carried out in controlled and uncontrolled ways.

	Presence of Side-Reactions with Δ*S* > 0	Overall Entropy Change	Thermodynamic Spontaneity
Controlled processes	no	Δ*S_tot_* = Δ*S_r_* < 0	*T* < T* = Δ*S_r_*/Δ*H*
Uncontrolled processes	yes	Δ*S_r_* < Δ*S_tot_* < 0	*T* < Δ*S_tot_*/Δ*H*
		Δ*S_tot_* > 0	For every T

## Data Availability

Data are contained within the article.

## Nomenclature

Latin letters	
*A*	Helmholtz free energy
*E*	Energy content of a system
*G*	Gibbs free energy
*H*	Enthalpy
*L*	Work exchanged
*Lc*	Work exchanged in a closed system
*Lo*	Work exchanged in an open system
*M*	Molecular weight
*n*	Number of moles
*p*	Pressure
*Q*	Heat exchanged
*Q_p_*	Heat exchanged at constant pressure in reversible processes
*Q_V_*	Heat exchanged at constant volume in reversible processes
*Q_value_*	Energy released by a nuclear process
*R*	Gas constant
*S*	Entropy
*S_i_*	Entropy of irreversible processes
*T*	Temperature
*T**	Critical temperature
*U*	Internal energy
*V*	Volume
Greek letters	
*δ*	Non-exact differential
*μ* *_i_*	Chemical potential of the component “i”
Subscripts	
*chem*	Chemical reaction
*id*	Perfect gas
*nuc*	Nuclear reaction
*plasma*	Plasma, ionized gas
*sur*	Surroundings
*univ*	Universe, i.e., the union of the open system and its surroundings
